# 612 A Novel Antimicrobial Bioresorbable Matrix is Compatible With A Collagen And Glycosaminoglycan Bioresorbable Wound Matrix

**DOI:** 10.1093/jbcr/irac012.240

**Published:** 2022-03-23

**Authors:** Michael Schurr

**Affiliations:** MAHEC, Fairview NC, North Carolina

## Abstract

**Introduction:**

Skin substitutes are indicated for clean wounds because their use in colonized or infected wounds can lead to non-adherence, loss of graft, and subsequent waste of resources. Wound colonization can be hard to detect, and skin substitutes can trap microbes and potentiate wound infection. Therefore, the aim of this study was to evaluate the compatibility of a novel bioresorbable antimicrobial wound matrix containing low levels of silver (Silver Matrix), with a widely used skin substitute composed of a crosslinked bovine tendon collagen and glycosaminoglycan matrix (Collagen Matrix), to assess effects on wound healing progression and bioburden management.

**Methods:**

Clean and contaminated full-thickness wounds were made using a porcine model, and treated with either Silver Matrix or Collagen Matrix. Treatment groups included: 1) Clean treated with Silver and Collagen Matrix, 2) Clean treated with Collagen Matrix only, 3) Contaminated treated with Silver and Collagen Matrix, and 4) Contaminated treated with Collagen Matrix only. The Silver Matrix was applied directly to the wound bed, followed by the Collagen Matrix. Clean wounds were analyzed 14 and 27 days after treatment to evaluate wound healing progression as evidenced by angiogenesis, re‐epithelialization, inflammation, and fibroplasia. Contaminated wounds were analyzed 5 days after treatment to measure bacteria reduction, as evidenced by colony forming units (CFUs).

**Results:**

At day 14, angiogenesis, re‐epithelialization and fibroplasia were insignificantly different, and inflammation markers were modestly higher in the Silver and Collagen Matrix group. At day 27, there were no statistically significant differences in these metrics between groups. At day 5, infected wounds treated with Silver and Collagen matrix showed a 2-log reduction in CFUs, whereas wounds treated with Collagen Matrix only did not show a reduction.

**Conclusions:**

Covering full-thickness porcine wounds with a bioresorbable Silver Matrix before application of the skin substitute Collagen Matrix resulted in a significant decrease in bioburden without impairing wound healing progression.

